# Probing the Demulsification Mechanism of Emulsion with SPAN Series Based on the Effect of Solid Phase Particles

**DOI:** 10.3390/molecules28073261

**Published:** 2023-04-06

**Authors:** Qingchao Cheng, Guangsheng Cao, Yujie Bai, Zhixuan Zhu, Ning Zhang, Dongju Li

**Affiliations:** 1 Key Laboratory of Enhanced Oil & Gas Recovery of Ministry of Education, Northeast Petroleum University, Daqing 163318, China; 2 Research Institute of Oil Production Engineering, PetroChina Daqing Oilfield Limited Company, Daqing 163453, China; 3 Heilongjiang Provincial Key Laboratory of Oil and Gas Reservoir Stimulation, Daqing 163453, China

**Keywords:** non-ionic surfactant, molecular simulation, demulsification, nanoparticles, electric dehydration

## Abstract

The solid particles in the produced fluids from the oil wells treated by compound flooding can greatly stabilize the strength of the interfacial film and enhance the stability of the emulsion, increasing the difficulty of processing these produced fluids on the ground. In this paper, the oil phase and the water phase were separated from the SPAN series emulsions by electrical dehydration technology and adding demulsifier agents. The changing trends of the current at both ends of the electrodes were recorded during the process. The efficient demulsification of the emulsion containing solid particles was studied from the perspective of oil-water separation mechanisms. Combined with the method of molecular dynamics simulation, the effect of the addition of a demulsifier on the free movement characteristics of crude oil molecules at the position of the liquid film of the emulsion were further analyzed. The results indicated that the presence of solid particles greatly increased the emulsifying ability of the emulsion and reduced its size. Under the synergistic effect of demulsifier and electric dehydration, the demulsification effect of the emulsion increased significantly, and the demulsification rate could reach more than 82%. The addition of demulsifiers changed the stable surface state of the solid particles. The free movement ability of the surrounding crude oil molecules was enhanced, which led to a decrease in the strength of the emulsion film so that the water droplets in the emulsions were more likely to coalesce and break. These results are of great significance for the efficient treatment of wastewater from oilfields, promoting the sustainability of environment-friendly oilfield development.

## 1. Introduction

With the gradual development of the continental oilfields, the old area oilfields adopted the compound oil-driven system with polymer, alkali, and surfactant as the main components. The compound system (alkali surfactant (AS), surfactant polymer (SP), alkali polymer (AP), and alkali surfactant polymer (ASP) flooding system) can make great contributions to improving oil recovery [1]. However, the application of the compound system has led to some problems. The produced fluids of the compound flooding system may cause scaling and the phenomenon of seriously blocking the formation [2]. The hydrochloric acid system and the hydrofluoric acid system, as low-cost acidification working fluids, can effectively remove the problem of scaling in the formation [3]. Nevertheless, it was seen from the perspective of the on-site construction results that the emulsification problem of produced fluids from acidified oil wells was more serious [4]. This could be explained by the fact that the polymer, surfactant, and nanoparticles formed after acidification enabled the produced fluids to form the Pickering emulsion, thus enhancing the degree of emulsification of the emulsion [5]. The emulsification of crude oil led to an increase in the difficulty of processing the produced fluids on the ground. Difficulties in the oil-water separation of produced fluids can increase field development expenses [6]. Consequently, the demulsification of emulsion with solid particles has been one of the key issues of produced water treatment in oil fields so far.

**Figure 1:**
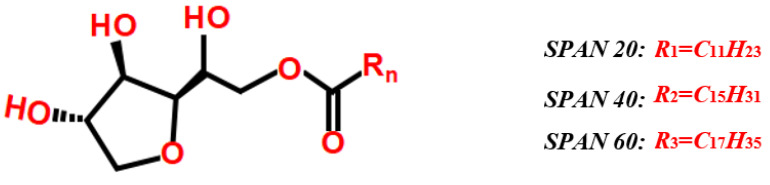
Molecular structure of the SPAN series surfactant.

The non-ion surfactants of the SPAN series are commonly used in oilfield chemical flooding, including SPAN 20 (sorbitan monolaurate, C_18_H_34_O_6_), SPAN 40 (sorbitan monopalmitate, C_22_H_42_O_6_), SPAN 60 (sorbitan monostearate, C_24_H_46_O_6_), and so on. As demonstrated in Figure 1. The difference in the molecular structure of SPAN 20, SPAN 40, and SPAN 60 is the length of the carbon chain in *R*_n_.

Many studies have reported the oil displacement efficiency of SPAN series surfactants [7,8,9]. Former studies have shown that SPAN series non-ionic surfactants had excellent thermal stability and were suitable for high-temperature reservoir conditions [10]. The combination of SPAN 60 and Tween 80 with sodium hydroxide can reduce the interface tension between oil and water to emulsify crude oil in the formation water [11]. Moreover, the addition of SPAN series surfactants and hydrophobic SiO_2_ nanoparticles has a great effect on the characteristics of the interface between crude oil and water. The enhanced oil recovery potential of microemulsion systems containing nanoparticles with different concentrations has been investigated through core experiments [12]. The emulsion with solid phase particles is extremely stable, and it is necessary to effectively separate oil and water from the emulsion containing solid phase particles. The commonly used oil-water separation technology is mainly divided into two aspects, including physical methods and chemical methods. Physical oil-water separation methods include gravity separation, cyclone separation, post-mixing filtration separation, electric separation, heating separation technology, etc. [13,14,15,16,17]. The chemical method refers to separating oil and water by adding a demulsifier to the emulsion [18]. The chemical demulsification method has been well recognized as one of the effective methods for oil-water separation in a highly stable emulsion [19,20,21]. However, a single physical or chemical method is not able to meet the requirements of the large-scale treatment fluid. The combination separation method has become an important choice. When the ultrasonic treatment technology is combined with additives, the compound means have different demulsification abilities for different oil in water emulsions. Ultrasonic parameters such as power and time have a great impact on the separation efficiency of emulsions in intermittent mode [22]. Although an ultrasonic treatment device has a relatively strong ability to demulsify, the device only works at a limited distance. For emulsions containing solid particles, the dispersion degree of solid particles is enhanced in the range where the ultrasonic wave sweep effect is weak. The separation efficiency achieved by this method is low, and the application effects in the field are poor. Considering the ability of the fiber material to intercept and capture the water droplets, a treatment method involving a DC electric field and medium convergence was proposed, which greatly improves the demulsification efficiency [23]. A demulsification dewatering device coupled with a high-voltage electric field and cyclone centrifugal field can realize that the dehydration rate and oil removal rate increase by 15.3% and 12.4%, respectively, when the voltage increases from 0 to 11 kV [24]. Therefore, electric dehydration technology has strong advantages in the efficient treatment of oil-water emulsions. Many studies have been carried out on the mechanism of demulsification [25,26,27,28,29,30]. Acidizing has been applied to remove skin damage from the oil wells flooded by ASP. However, few studies have been conducted on the effect of solid particles formed in this process on the stability of the emulsion of the fluids produced from these acidizing wells. One of the reasons for the strong emulsifying stability of the emulsion is the synergistic effect among the ferrous sulfide (FeS) nanoparticles produced by the formation, the SiO_2_ particles dispersed from the formation water, and the surfactants [31]. SiO_2_ particles have the highest content in solid particles, followed by FeS and CaCO_3_ particles. Nevertheless, previously, there was a lack of studies on the effects of solid particles and demulsifiers on the stability of emulsions.

The oil-water separation mechanism of an emulsion containing solid particles under the effect of a demulsifier has not been elucidated. The demulsification effect of the SPAN series emulsion has not been studied comprehensively by scholars. In this paper, the stability of SPAN series emulsions containing solid particles was analyzed from the perspective of the separation mechanism between the oil phase and the water phase in an emulsion containing solid particles. The electric dehydration method was used for the demulsification of SPAN series emulsions. The change trends of the current at both ends of the electrode in the process of separation were recorded to analyze the effect of solid nanoparticle type on the current in the electrical dehydration process. A demulsifier agent was added to the emulsion with solid particles to realize efficient separation. Combined with the molecular dynamics simulation, the effect of the demulsifier on the free movement characteristics of crude oil molecules at the location of the emulsion liquid film was further analyzed. The optimized theory of oil-water separation to boost demulsification efficiency was proposed to improve the treatment efficiency of oilfield-produced fluids, thereby providing guidance for efficient treatment of the produced fluids from oilfields flooded by compound systems.

## 2. Results and Discussion

### 2.1. Characteristics of Oil-Water Emulsion with Solid Particles

**Figure 2:**
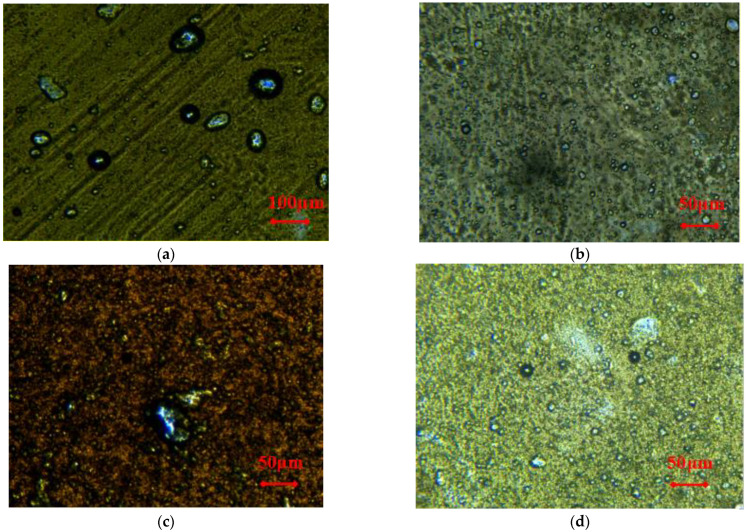
Oil-water emulsion systems with different types of emulsifiers. (**a**) Emulsion with SPAN 20 as an emulsifier; (**b**) Emulsion with the combination of SPAN 20 and SiO_2_ nanoparticles as an emulsifier; (**c**) Emulsion with the combination of SPAN 40 and SiO_2_ nanoparticles as an emulsifier.; (**d**) Emulsion with the combination of SPAN 60 and SiO_2_ nanoparticles as an emulsifier.

The content of SiO_2_ particles in the produced fluid was relatively high; as a result, the characteristics of an oil-water emulsion containing SiO_2_ nanoparticles were studied. The emulsifying phenomena of the oil-water emulsion systems with different types of emulsifiers were observed under the microscope. Figure 2 illustrates that the states of the emulsions formed by adding different combinations of emulsifier systems and solid nanoparticles were quite different. As shown in Figure 2a, when the single SPAN 20 was used as the emulsifier, the emulsifying degree of the system was relatively low; only a small amount of emulsion particles existed, and these emulsion particles were relatively large in size. Compared with the microtopography illustrated in Figure 2b–d, it was found that when both solid particles and emulsifiers were added to the emulsion, the degree of emulsification was greatly increased and several emulsion particles with small sizes were formed.

The characteristics of the emulsion systems changed when different combinations of emulsifier systems and solid nanoparticles were added. The particle size distribution and stability of emulsions with different emulsifier combinations are shown in Table 1. The emulsifying degree of the emulsion system increased obviously when using a mixture of nanoparticles and a series of SPAN chemical agents as emulsifiers. While there was an obvious reduction in the size of the emulsion particles, the maximum particle size of the emulsion decreased from 255.7 μm to 3.11 μm, and the minimum particle size was 0.51 μm. The emulsifying system presented a relatively uniform state, which could be attributed to the substantial enhancement of the emulsifying ability of solid nanoparticles on the emulsion. Meanwhile, the demulsification rate changed with demulsification time. The addition of solid nanoparticles could enhance the stability of the emulsion systems significantly. By comparing the properties of the emulsion systems with the addition of different emulsifiers, it was found that the particle size of the emulsion did not change significantly with the increase in the length of the carbon chain. While with the increase in the length of the carbon chain of the emulsifier, the emulsification stability of the emulsion was gradually enhanced.

**Figure 3:**
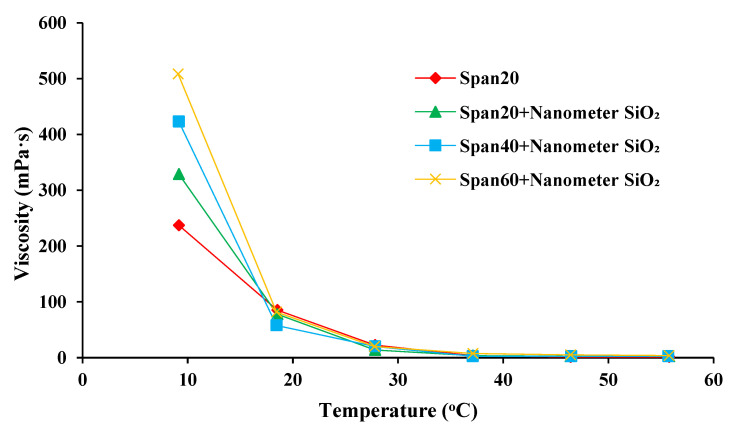
Oil-water emulsion systems with different types of emulsifiers.

The viscosities of the emulsion systems with different emulsifier combinations of SPAN systems and solid nanoparticles were further measured. Figure 3 demonstrates that the viscosity of the emulsion increased significantly after the addition of nanoparticles. As the length of the carbon chain increased, the viscosity of the emulsion changed a little. The results were consistent with those of emulsion stability. Under the condition of low temperature, the viscosity of the emulsion was up to 510 mPa·s. When the temperature of the system reached more than 50 °C, the viscosity of the emulsion gradually decreased, and the lowest viscosity was 15 mPa·s. Hence, the emulsion system could maintain its stability under low-temperature conditions, while it was prone to demulsify when the emulsion was placed in an environment with a high temperature.

### 2.2. Demulsification Laws of SPAN Series Emulsions Containing Solid Particles under a High-Voltage Electric Field

**Figure 4:**
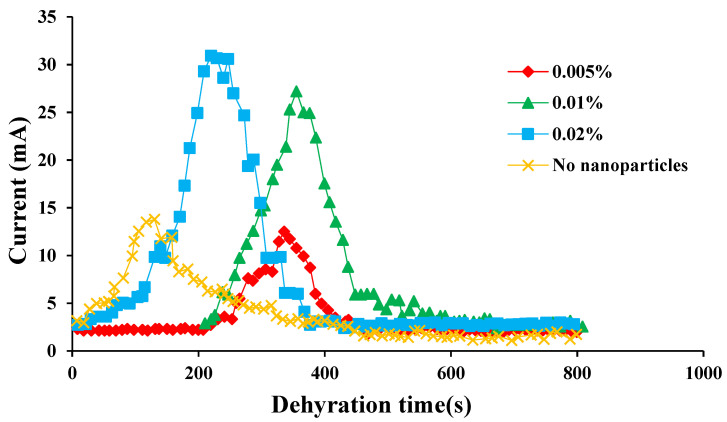
The current variation of the emulsion with the addition of different concentrations of CaCO_3_ nanoparticles with time when the voltage was 1500 V.

**Figure 5:**
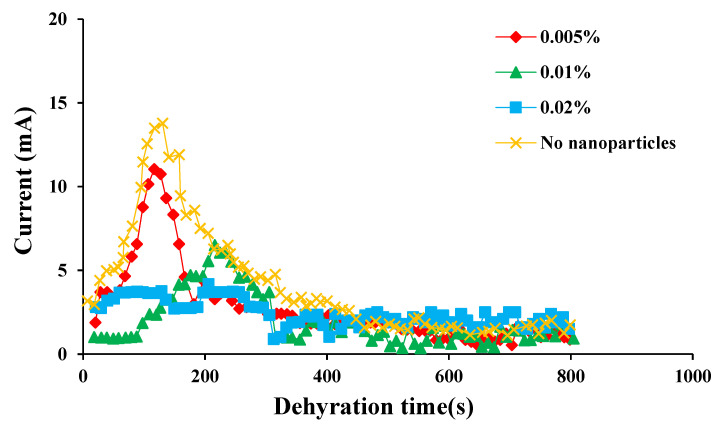
The current variation of the emulsion with the addition of different concentrations of SiO_2_ nanoparticles with time when the voltage was 1500 V.

**Figure 6:**
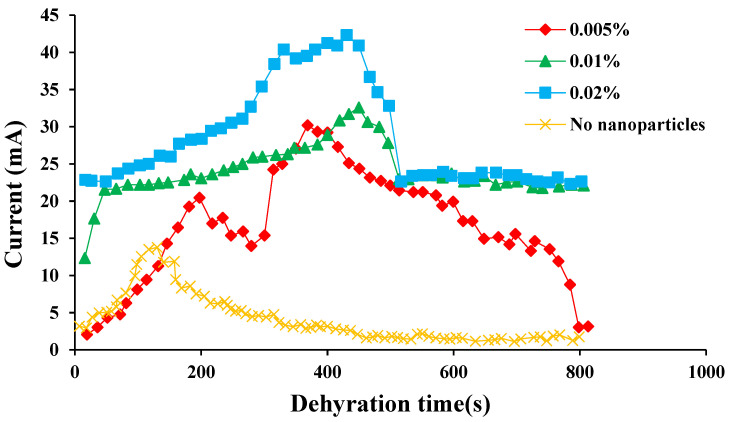
The current variation of the emulsion with the addition of different concentrations of FeS nanoparticles with time when the voltage was 1500 V.

In the process of electric dehydration, the variation of the current at both ends of the electrode was used to characterize the separation process. The changes in current at both electrodes during the electric dehydration process of the SPAN 20 emulsion systems with different nanoparticles as emulsifiers are shown in Figure 4, Figure 5 and Figure 6.

In Figure 4, it is shown that when the concentration of CaCO_3_ nanoparticles was 0.02%, the current of the emulsion changed in the first 100 s of electric dehydration, which meant that the demulsification was beginning. The peak value of current appeared when the electric dehydration time was between 200 and 250 s, indicating that the demulsification speed of the emulsion reached its peak in this time interval. While the concentration of CaCO_3_ nanoparticles was low (between 0.005% and 0.01%), the current began to change at about 200 s, and the emulsion began to demulsify. The current peak value increased with the increase in the concentration of CaCO_3_ nanoparticles. Further, emulsions without nanoparticles demonstrated shorter demulsification times. It could be interpreted as the fact that the addition of CaCO_3_ nanoparticles enhanced the emulsification effect of the emulsion, and made the dispersed phase droplets charge and have strong electrical conductivity. The deformed water droplets could coalesce, collide with each other, merge under the action of electrophoresis, and then settle out of the emulsion.

As shown in Figure 5, the peak current gradually decreased with the concentration of SiO_2_ nanoparticles, indicating that the emulsifying ability of the emulsion was stronger. The variation law of the peak current of the emulsion containing SiO_2_ nanoparticles in the process of electrical dehydration was completely opposite to that of the emulsion containing CaCO_3_ nanoparticles. Moreover, the measured maximum current of the emulsion containing SiO_2_ nanoparticles was smaller than that of the emulsion containing CaCO_3_ particles, which also proved that the demulsification rate of the emulsion containing SiO_2_ nanoparticles was slower. This difference was caused by the non-electability of SiO_2_ nanoparticles. SiO_2_ nanoparticles were mainly adsorbed on the oil-water interface film to prevent the demulsification of the emulsion.

The effects of the concentration of FeS nanoparticles on the electrical properties of emulsions are shown in Figure 6. The conductivity of the emulsion system with FeS nanoparticles was high. During the process of electrical dehydration, the initial current changed significantly. The peak current increased with the increase in FeS nanoparticle concentration, reaching 42.5 mA. Hence, the addition of FeS nanoparticles could increase the charge capacity of the liquid droplets in the dispersion and enhance the conductivity of the emulsion. When the concentration of FeS nanoparticles was greater than 0.05%, a cross-electric field phenomenon occurred, resulting in an inability to achieve the separation of oil and water under the condition of electrical dehydration.

Further, 0.2% sodium dodecyl sulfate was added to the emulsion to achieve demulsification. Then, the current and demulsification situation of the formed emulsion with the combination of a series of SPAN agents and solid particles as emulsifiers in the process of electrical dehydration were measured to further analyze the demulsification effect under the synergic action of surfactant and solid particles.

The results illustrated in Table 2 show that the effect of electrical dehydration was significantly enhanced after adding a demulsifier to the emulsion with solid nanoparticles. The maximum current during the process of electrical dehydration technology was reduced, and the minimum current was maintained at about 0.1 mA. The demulsification rate remained above 82%, and the highest demulsification rate was 96.22%. Therefore, the conclusion that the physical action of electric dehydration and the chemical action of the surfactant system had a synergistic effect in the demulsification process of emulsion, thus reducing the occurrence of cross-field phenomena and enhancing the dehydration rate, was drawn.

### 2.3. Demulsification Mechanism of Emulsion with Solid Particles

**Figure 7:**
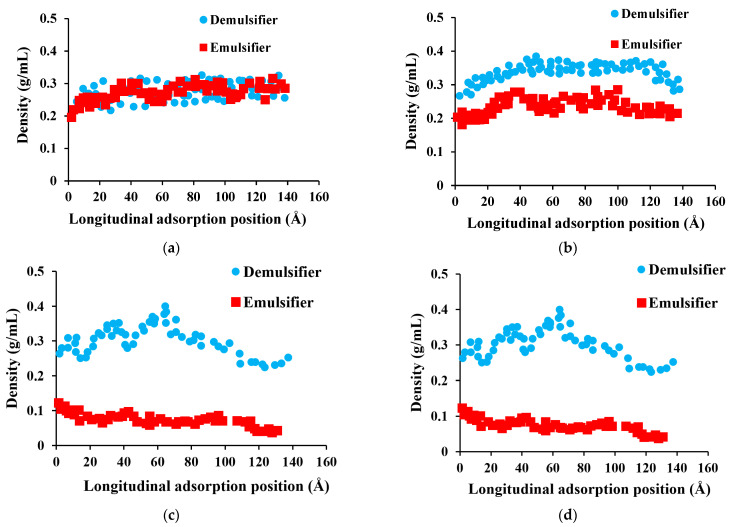
Characteristics of competitive adsorption between the demulsifier and emulsifier on the SiO_2_ surface at different times, (**a**) 20 ps; (**b**) 100 ps; (**c**) 300 ps; (**d**) 1000 ps.

The molecular dynamics method was used to further analyze the demulsification mechanism of an emulsion with solid particles under the action of the demulsifier. The density distribution of the demulsifier and emulsifier in the system at different times was calculated according to the molecular model and simulation method described in Section 2.2. The characteristics of competitive adsorption between demulsifier (sodium lauryl sulfate) and emulsifier (SPAN 20) on the SiO_2_ surface at different times are illustrated in Figure 7. There are major differences between the density distribution characteristics of the demulsifier and emulsifier at different times. At the initial moment, the density distribution of the demulsifier and emulsifier on the silica surface was relatively uniform. With the increase in simulation time, the density of the emulsifier on the nano-silica surface gradually decreased, indicating that competitive adsorption between the emulsifier and demulsifier on the silica surface occurred. The demulsifier had a stronger adsorption capacity on the nano-silica than the emulsifier, which stripped the emulsifier from the rock surface. Furthermore, the synergistic emulsification effect of silica solid and the emulsifier was affected, and the control ability of silica solid particles on the emulsion film was reduced. It is seen from Figure 7d that when the simulation time reached 1000 ps, the emulsifier density on the nano-silica surface was about 0.1 g/mL, indicating that the adsorption replacement of the demulsifier had completed at this time and the effect of the emulsifier decreased greatly. The addition of a demulsifier could weaken the strength of the interfacial film and increase the diffusion coefficient of oil molecules at the oil film position, thus making the oil film more prone to rupture.

**Figure 8:**
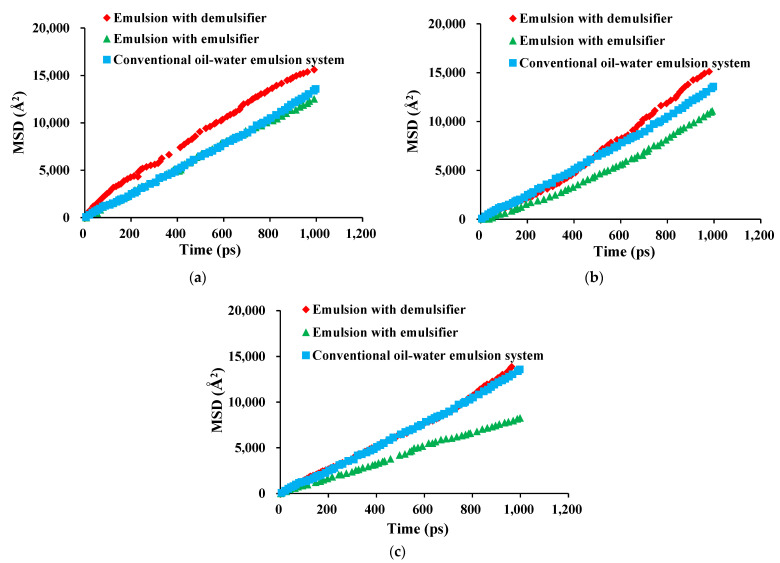
Change in root mean square displacement of crude oil molecules at liquid film position with time. (**a**) Root mean square displacement of emulsion before and after demulsification with SPAN 20 as an emulsifier. (**b**) Root mean square displacement of emulsion before and after demulsification with SPAN 40 as an emulsifier. (**c**) Root mean square displacement of emulsion before and after demulsification with SPAN 60 as an emulsifier.

In order to explore whether the change in the diffusion coefficient of crude oil molecules led to the easier destruction of the oil film, the root mean square displacement was calculated. The root mean square displacement (MSD) represented the free motion ability of crude oil molecules at the liquid film position, and the larger the slope, the stronger the free motion ability. As shown in Figure 8, the MSD curve of a conventional emulsion system without nanoparticles was set as the blank control group for comparison. The variation of MSD values of crude oil molecules at the position of the liquid film in the SPAN series emulsion liquid system over time before and after the addition of demulsifiers were calculated, respectively. The MSD value of crude oil molecules at the position of the emulsion liquid film of different emulsifiers changed significantly before and after demulsification. When the emulsifier SPAN was added, the slope of the curve of the MSD of the crude oil molecule changing with time decreased significantly. It was also observed that the longer the carbon chain length of the SPAN surfactant contained in the emulsion, the stronger the inhibition on the free movement of crude oil molecules. Hence, it was concluded that the emulsifier could effectively inhibit the free movement ability of crude oil molecules and enhance the strength of the liquid film, thereby enhancing the stability of the emulsion. However, the addition of a demulsifier increased the slope of the curve of the MSD of crude oil molecules changing with time because, after the replacement of the demulsifier on the surface of nano-silica, the free movement ability of crude oil molecules at the liquid film position was enhanced, which greatly reduced the strength of the liquid film and made the emulsion liquid film more prone to being broken.

**Figure 9:**
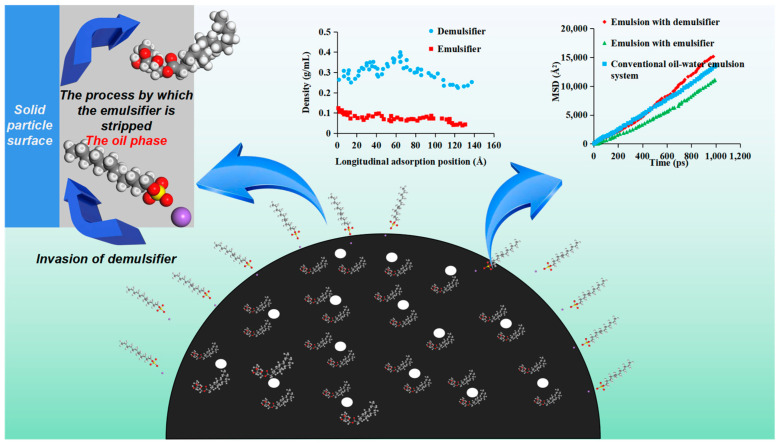
Schematic diagram of the demulsification mechanism of emulsion containing SPAN series surfactant under the action of demulsifier and solid particles.

Consequently, the demulsification mechanism of an emulsion with SPAN and solid particles can be drawn based on the results of the above experiments and simulations, as shown in Figure 9. For emulsion systems containing solid particles, a certain amount of emulsifier was adsorbed on the surface of the solid particles. The solid particles cooperate with the emulsifier (water-in-oil) to inhibit the free movement ability of the crude oil molecules at the position of the emulsion liquid film, thereby enhancing the strength of the liquid film. When a demulsifier (oil-in-water) is added to the emulsion, it plays the role of peeling off the emulsifier system originally adsorbed on the surface of solid particles, changing the original stable state. Moreover, after the demulsifier is adsorbed on the surface of the solid particles, it can enhance the free movement ability of the surrounding crude oil molecules, thereby reducing the strength of the emulsion liquid film and making the emulsion easier to coalesce and break.

## 3. Materials and Methods

### 3.1. Experimental Section

#### 3.1.1. Experimental Materials and Devices

Emulsifiers SPAN 20 (98%), SPAN 40 (98.5%), and SPAN 60 (98%) were all produced by Daqing Xuanye Chemical Co., Ltd. The crude oil was provided by Daqing Oilfield No. 1 Oil Production Plant. Calcium carbonate (CaCO_3_) nanoparticles with a diameter of 25 nm were produced by Shanghai Chenqi Chemical Technology Co., Ltd. Silica (SiO_2_) nanoparticles with a diameter of 20 nm were produced by Shanghai Steel Metallurgy Co., Ltd. Ferrous sulfide (FeS) nanoparticles with a median particle size of 30 nm were separated from the produced fluids of oil wells in the Daqing Oilfield No. 1 Oil Production Plant. Ultrapure water was used to carry out the experiments.

The high-speed shearing dispersion emulsifying machine (FA25) for preparing emulsion was produced by Fluko Chemical Co., Ltd. in Shanghai, China. The emulsion particles were observed using a PM 6000 electron microscope made by Hengqin Instrument and Equipment Factory in Shanghai, China. The Brookfield electronic viscometer (DV3) was used to measure the viscosity of the emulsion manufactured by Massachusetts, USA. Electric dehydration and demulsification were carried out with the DPY-2 demulsifier selection apparatus produced by Jiangyan Instrument Co., Ltd., Taizhou, Jiangsu Province, China.

#### 3.1.2. Preparation and Stability Evaluation of Emulsion with Solid Particles

The emulsion systems with solid particles were prepared by following the next steps. The analytical balance was used to weigh ultrapure water and nanoparticles. First, 20 mL of crude oil was weighed and placed into a beaker; simultaneously, 20 mL of ultrapure water was measured. The beakers with the two liquids were preheated in a water bath at 50 °C. The 0.1 g of solid nanoparticle was added to the crude oil sample at a temperature of 50 °C. The oil samples were stirred by a high-speed shearing dispersion emulsifying machine at a speed of 10,000 r/min for 5 min so that the nanoparticles could be fully dispersed in the oil sample. The water sample was mixed with the crude oil containing nanoparticles, and then 0.2 g of emulsifier was quickly added to the not-fully-prepared emulsion. The emulsifying machine was used to emulsify and shear the emulsion at a high speed for 30 s to obtain a stable water-in-oil emulsion system with solid particles.

The morphology of the stable emulsion was observed by an electron microscope. The change laws and distribution of the size of the emulsion with solid particles were tested by the image analysis software. Then, the prepared emulsion was put into the test tube with scale lines to measure the demulsification degree of the emulsion at different times and calculate the demulsification rate.

Since the viscosity of water in an oil emulsion could reflect its emulsification characteristics, the viscosity of the emulsion with solid particles was measured. The temperatures of the emulsion systems were adjusted between 10 °C and 60 °C, and an electronic viscometer was used to measure the viscosity change in the emulsion systems.

#### 3.1.3. Demulsification Laws of Emulsion with Solid Particles

**Figure 10:**
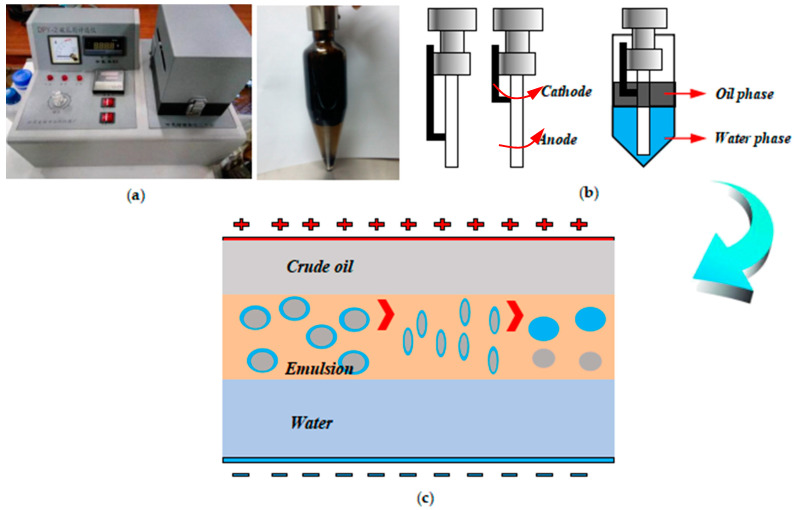
The device and the schematic diagram of the principle of the electric dehydration method. (**a**) Electric dehydration experimental device, (**b**) Working principle of electrode, (**c**) Demulsification principle of emulsion under electric field.

As shown in Figure 10a, the demulsifier selection apparatus had been widely used for the treatment of oilfield-produced fluids and had achieved good demulsification effects. When using this apparatus, the demulsification rates of conventionally produced fluids could always reach a value of over 90%. Therefore, the demulsification rules of emulsion systems were investigated by this device to study the movement states of emulsion particles during the demulsification process of emulsions containing solid particles. The main function of the demulsifier selection apparatus in the laboratory was to analyze the current change characteristics at both ends of the different emulsion systems under the action of an electric field, and hence, the demulsification state of the emulsions could be explored.

The DC electrodes were quickly inserted into the freshly prepared emulsion, and the electric field separator was used. The positive electrode was placed in the oil phase of the emulsion, while the negative electrode was in the water phase. The electric field separator with the prepared crude oil emulsion was placed into the aluminum heater, whose temperature was set at 50 °C in advance. Then electric dehydration was applied to the crude oil emulsion with solid particles at proper voltage values of 1500 V, which was commonly used in field sites. The water droplets of the emulsion would approach and coalesce gradually due to the action of the electric field; as a result, the oil phase and the water phase separated to realize the demulsification effect. The current values during the electrical dehydration process were recorded in real time. The demulsification rate of the emulsion with solid particles was calculated by taking out the electric field separator every 5 min.

### 3.2. Molecular Dynamics Simulation

#### 3.2.1. Establishment of the Molecular Model for Competitive Adsorption between the Demulsifier and Emulsifier

**Figure 11:**
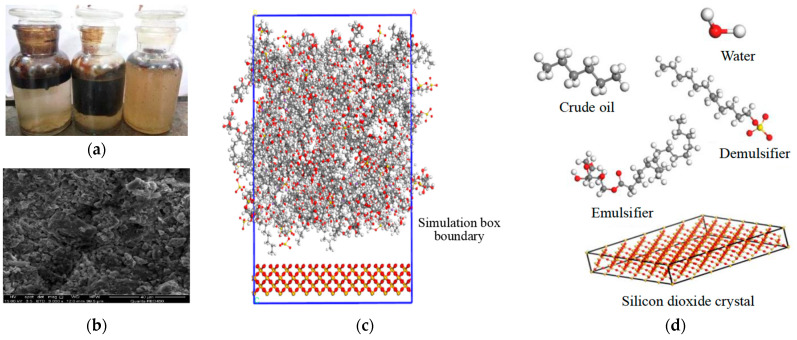
The schematic diagram of the competitive adsorption model of different molecules of emulsifiers and demulsifiers on the surface of solid particles. (**a**) The state of the oil-water emulsion after separation, (**b**) Electron microscopic image of solid particles, (**c**) Competitive adsorption model on the surface of solid particles, (**d**) Competitive adsorption model for competitive adsorption between the demulsifier and emulsifier.

Many studies have shown that solid particles have a relatively great impact on the stability of emulsions. A molecular dynamics model for competitive adsorption between the emulsifiers and demulsifiers was established to simulate the competitive adsorption process of the emulsifiers and demulsifiers on the surface of nano-silica. As shown in Figure 11, the solid particles had a strong adsorption capacity on the oil phase; as a result, the oil phase was selected as the dispersion phase in the molecular model. The competitive adsorption model of different molecules of emulsifiers and demulsifiers on the surface of nano-silica is illustrated in Figure 11d. The competitive adsorption process of emulsifiers and demulsifiers on the surface of nano-silica in the oil phase was investigated. In addition, considering the complex composition of the actual crude oil, the low-carbon components such as methane and ethane were mainly presented as the gas phase in the formation. Therefore, C6 was selected as the crude oil component in the model, and the molecular number of C6 was set at 500. Both the amount of emulsifier SPAN 20 and the amount of demulsifier sodium dodecane sulfate were set to 20.

#### 3.2.2. Simulation Methods

The initial model was obtained by performing an overall minimization operation on the energy. The intermolecular interaction potential of the initial model was the Lennard–Jones potential with a truncation radius of 12.5 Å. The long-range Coulomb force was calculated by the PPPM algorithm. After constructing the initial model, the shake method was used to make the crude oil component shake near its geometric position and fix the geometric position of the crude oil component. The system was dynamically simulated in an NVT ensemble for 1 ns. The Nose–Hoover thermostat was used to control the system temperature at 323.15 K with a time interval of 0.1 ps.

## 4. Conclusions

In this study, the demulsification mechanism of oil-water emulsion containing nanoparticles was analyzed. The separation characteristics of oil-water emulsions containing nanoparticles were studied by combining the results of the electrical dehydration experiment with the molecular dynamics method. The results showed that the stability of the emulsion was greatly enhanced after the addition of nanoparticles. The size of the formed liquid droplets in the emulsion could not change significantly with the increase in the length of the carbon chain of the series of SPAN emulsifiers, while the emulsifying stability was gradually enhanced with the increase in the length of the carbon chain of the emulsifier. The demulsifier could replace the emulsifier adsorbed on the surface of solid particles, changing the original stable state. According to the results of this paper, for emulsions containing nanoparticles, a demulsifier can be used to change the solid particle surface and the stable state of the oil-water interfacial film, so that water droplets in the emulsion are more likely to coalesce. These studies are of great significance for improving the oil-water separation theory of emulsion, so as to improve the treatment efficiency of oilfield-produced liquid and provide guidance for environmental protection in the process of oil exploitation and development.

## Figures and Tables

**Table 1:** The particle size distribution and stability of emulsions with different emulsifier combinations.

Number of the Sample	Emulsion Composition			Size of Emulsion Particles (μm)			Demulsification Rate (%)			
	Type of Surfactant	Surfactant Concentration (%)	Quality of SiO_2_ Nanoparticles (g)	Maximum Particle Size	Minimum Particle Size	Average Particle Size	5 min	10 min	20 min	60 min
1	SPAN 20	0.2	0	255.7	61.5	119.73	95.32	81.22	68.12	41.25
2	SPAN 20	0.2	0.1	3.11	0.51	1.41	98.61	96.34	90.28	77.42
3	SPAN 40	0.2	0.1	3.62	0.52	1.29	98.32	96.76	93.51	84.81
4	SPAN 60	0.2	0.1	3.27	0.59	1.22	98.11	96.51	94.65	87.11

**Table 2:** Electrical dehydration properties of emulsion with the combination of surfactant and solid particles as an emulsifier.

Number	Surfactant		Solid Particles		Maximum Current (mA)	Minimum Current (mA)	Initial Demulsification Time (s)	Final Demulsification Rate (%)
	Type	Concentration	Type	Concentration				
1	SPAN 20	0.2%	CaCO_3_ nano- particles	0.01%	4.32	0.11	122	92.14
2	SPAN 40				5.74	0.13	62	90.25
3	SPAN 60				8.17	0.11	11	87.34
4	SPAN 20		SiO_2_ nano- particles		3.14	0.14	177	96.14
5	SPAN 40				4.25	0.12	84	96.22
6	SPAN 60				7.66	0.11	23	95.33
7	SPAN 20		FeS nano- particles		15.68	0.11	0	87.11
8	SPAN 40				21.34	0.12	0	85.51
9	SPAN 60				25.11	0.11	0	82.34

## Data Availability

The data presented in this study are available on request from the corresponding author.

## Sample Availability

Samples of the compounds are not available from the authors.
